# Supplementary material for: Testing both affordability-availability and psychological-coping mechanisms underlying changes in alcohol use during the COVID-19 pandemic
Source: PLoS One. 2022 Mar 24;17(3):e0265145. doi: 10.1371/journal.pone.0265145 (PMC8947385; doi:10.1371/journal.pone.0265145)
Supplement: Attachment — Submitted filename: Response to Reviewers.docx [file pone.0265145.s001.docx]

**PLOS One [***PONE-D-21-30816]*

**Response to Reviewers**

Reviewer #1: This is my second time reviewing this article. As I stated the first time around, there is a lot to like about this paper, including a large sample size, interesting and important questions, a great summary of prior work on changes in alcohol consumption as a result of the pandemic, and public access to the data. I offered the following suggestions to strengthen the paper when I reviewed this the first time, and the authors were highly responsive to all of my concerns in this updated version. I have one more question, though. Typically, there are four drinking motives, and I wondered why the authors combined social and enhancement? Overall, I think this paper makes a nice contribution to the literature.

--Prior concerns that were adequately addressed--
1. The paper would benefit from a figure or figures. There are a lot of variables and paths being tested and a figure would help in clarifying the models and results.
2. I had a hard time seeing how the four aims mapped onto testing the “psychological” and “affordability-availability” mechanisms. For instance, wouldn’t it be illuminating to test whether drinking to cope motives explain the link between increases in variables like depression, loneliness etc and increases in drinking behavior as a result of the pandemic? Also, did the authors control for other motives in their analyses to see unique effects? Finally, it was unclear why some of the variables were included (e.g., paranoia, personality traits). It would be helpful to better motivate these in the introduction.
3. I understand that there are 3 waves of data, but it was unclear when some of the measures were assessed (this info is omitted for many of the measures in the methods section). It would be helpful to clarify which measures were assessed when and, in the case where measures were collected at multiple time points, an explanation is needed for choosing one particular wave over another.
4. Relatedly to my point above, the authors don’t seem to take advantage of the longitudinal nature of the data in their analyses. The title of the paper and hypotheses focus on changes in alcohol use, but the analyses don’t account for changes in drinking behavior pre- to post-pandemic, or this wasn’t clear.
5. The sample is described as being nationally-representative on page 9, but on page 20 a limitation is that the results can’t be generalized to all adults. Are the authors able to clarify?
6. The small decrease in at risk drinking is described as being “slight” in the results section and the confidence intervals overlap, but the difference is described as being significant in some places of the text. Are the authors able to clarify this, too?
7. It’s unclear if you were able to account for individuals who stopped drinking as a result of the pandemic in analyses.
8. I thought scores on the AUDIT-C indicative of hazardous drinking varied for men (score of 4) and women (score of 3). The authors used a score of 5, but perhaps I’m not aware of this convention.

**AUTHORS’ Response:**

We thank the reviewer very much for taking the time to review our updated submission at PLOS One. We would like to thank them for their original review (Psychology of Addictive Behaviors). We carefully considered all the suggestions they raised at that time and revised the manuscript accordingly. We believe the manuscript has been improved as a result of incorporating these changes, so thank you.

With respect to the query about the combination of social and enhancement motives: we acknowledge the point the reviewer has made re the general assessment of four drinking motives. For instance, the Drinking Motives Questionnaire Revised (DMQ-R; Cooper et al., 1994; Kuntsche et al., 2006) comprises 20-items to assess social, coping, enhancement, and conformity motives in adolescence/young adulthood. The measure was developed for this developmental period but has been used to measure drinking motives in older drinkers (e.g., Gilson et al., 2013). During the design of the earliest waves of this on-going longitudinal survey during the first UK lockdown (spring-summer 2020), we wanted to administer questions to adult drinkers (aged 18 years +) that would capture aspects of their motives for drinking during a period of unprecedent change in their normal daily activities. We considered a range of established measures, but also examined the measures being used in other similar UK-based studies being conducted during lockdown (e.g., Irizar et al., 2021). Given concerns about alcohol being used primarily as a mechanism for self-medicating negative affect during this period of unprecedented change, we included more indicators of ‘drinking to cope’ to ensure that we would have a robust measure of this motive. This focus, coupled with survey resource constraints (i.e., length of questionnaire was constrained by financial resources and concerns about limiting respondent burden to maximise retention over time), meant we included three indicators of use of alcohol for social/enhancement purposes. This is a potential limitation of the study and we have included a note to this regard in the discussion of the revised paper (p.28).

*“A set of 15 statements was administered to measure drinking motives during the pandemic, but there was a particular focus on measuring drinking to cope motives given the unprecedented change to daily life activities during lockdown. Due to survey constraints (i.e., financial resources and in an attempt to minimise respondent burden), other traditionally assessed drinking motives - social, enhancement, and conformity motives - were measured using a small number of items (i.e., six items to measures these three constructs). Items assessing social and enhancement motives were combined to facilitate the specification and estimation of the confirmatory factor model,* *and this model provided a good fit to the data.”*

**References**

Cooper, M. L. (1994). Motivations for alcohol use among adolescents: Development and validation of a four-factor model. *Psychological Assessment*, 6(2), 117. <https://psycnet.apa.org/doi/10.1037/1040-3590.6.2.117>

Gilson, K.-M., Bryant, C., Bei, B., Komiti, A., Jackson, H., & Judd, F. (2013). Validation of the Drinking Motives Questionnaire (DMQ) in older adults. *Addictive Behaviors*, 38(5), 2196-2202. <https://doi.org/10.1016/j.addbeh.2013.01.021>

Irizar, P., Jones, A., Christiansen, P., Goodwin, L., Gage, S., Roberts, C., Knibb, G., Cooke, R., & Rose, A. K. (2021). Longitudinal associations with alcohol consumption during the first COVID-19 lockdown: associations with mood, drinking motives, context of drinking, and mental health. *Drug and Alcohol Dependence,* 108913. <https://doi.org/10.1016/j.drugalcdep.2021.108913>

Kuntsche, E., Knibbe, R., Gmel, G., & Engels, R. (2006). Replication and validation of the Drinking Motive Questionnaire Revised (DMQ-R, Cooper, 1994) among adolescents in Switzerland. European addiction research, 12(3), 161-168. <https://doi.org/10.1159/000092118>
